# 

**DOI:** 10.1192/bjb.2024.4

**Published:** 2024-08

**Authors:** Martin Curtice

**Affiliations:** is a consultant in old age psychiatry with the Older Adults Mental Health Service, Coventry and Warwickshire Partnership NHS Trust, Warwick, UK. Email: mjrc68@doctors.org.uk

**Figure:**
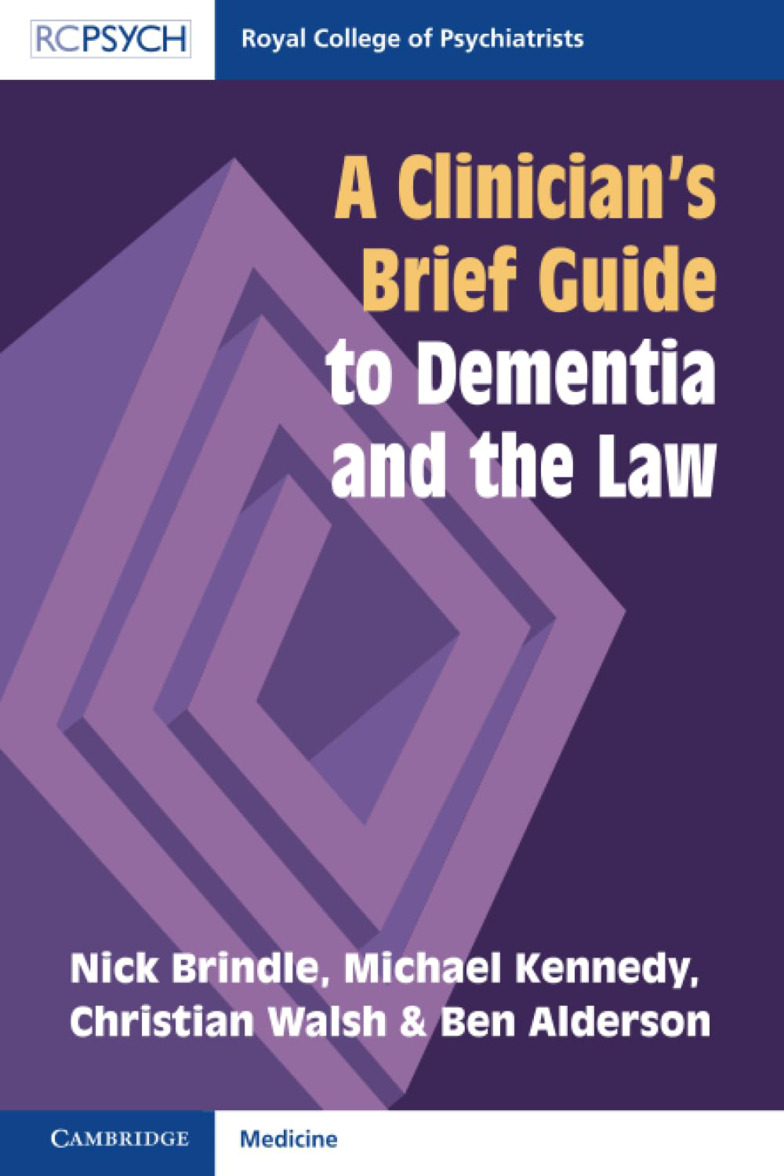

This is the fourth of the ‘clinician's brief guides to the law’ series, but the first that is diagnosis specific about dementia. It is designed to be, and is, practical and easy to read (only 150 pages, excluding appendices). It notes that areas of law relating to dementia care can be complex and difficult to navigate. The statutes most applicable in England and Wales are the Care Act 2014, Mental Capacity Act 2005 (MCA) and Mental Health Act 1983 (MHA). The authors acknowledge that, although resources in health and social care are ‘sorely depleted’, they believe having a good understanding of the law and rooting care ‘robustly’ in the legal framework has the potential to improve outcomes for individuals and carers. Additionally, healthcare professionals being able to show how law has been applied may provide ‘some insulation’ from criticism in the event of untoward incidents. The illustrative cases are all derived from court judgments.

The chapters on the MCA and MHA provide punchy overviews weaving in key practical aspects pertaining to people with dementia. The Care Act chapter is useful and notes that clinicians are less likely to be familiar with this Act compared with the MCA and MHA. The chapter on assessment of capacity is brilliant in how it contains so much practical advice on the many nuances of capacity assessments not just for people with dementia but in general.

Care and treatment legal issues for people with dementia are reviewed in various settings, including the home, care homes and hospitals. In doing so the book provides advice such as how to manage someone with dementia who refuses to accept community support, covert medication, end-of-life care and advance decisions to refuse treatment. Another chapter reviews legal aspects regarding discharge from hospital, which is especially useful for more complex cases, including the not uncommon and vexing issue of delayed discharges. The person with dementia in the context of the Mental Health Tribunal (MHA) and the Court of Protection (MCA) is reviewed. Practical advice includes advice on report writing for the Court of Protection (e.g. section 49 reports) and advice on assessing capacity for contact with others and consent to sexual relationships. Although a less common clinical arena, the book reviews the interface between dementia and the criminal justice system. It considers crimes committed against and by people with dementia and issues around people with dementia in prisons and secure hospitals.

This is undoubtedly an excellent and very readable book. It is packed with practical and sage advice. It covers an impressively comprehensive breadth of issues and is ideal for healthcare professionals working with people with dementia of any age. I'd suggest it is a must for care homes, community and in-patient teams to have a copy handily available. I sincerely hope this specialist book continues to be updated in years to come so as to provide expert support for healthcare professionals.

